# Innovative management program for achieving high yield and emission reduction: a case study of wheat/maize rotation production in the North China Plain

**DOI:** 10.3389/fpls.2026.1952966

**Published:** 2026-09-28

**Authors:** Yanchen Gong, Xiaoqin Jiang, Yongheng Du, Tengteng Liu, Ning Ma, Zhuyu Wang, Zhichao An, Jun Liang, Yanling Chen

**Affiliations:** 1 College of Resources and Environmental Sciences, Qingdao Agricultural University, Qingdao, China; 2 Research and Development Center, Wuzhoufeng Agricultural Science and Technology Co.,Ltd., Yantai, China; 3 Agricultural Comprehensive Service Center (Kongzhen), Laoling Agriculture and Rural Bureau, Laoling, China; 4 Shizhong Regional Agricultural Technology Extension Station, Laoling Agriculture and Rural Bureau, Laoling, China; 5 National Academy of Agriculture Green Development, Department of Plant Nutrition, College of Resources and Environmental Sciences, China Agricultural University, Beijing, China

**Keywords:** environmental costs, innovative management program, North China Plain, nutrient management, wheat–maize rotation

## Abstract

**Introduction:**

In the North China Plain, inadequate agronomic technology integration in the dominant wheat‒maize rotation causes yield loss, environmental risks, and low nutrient efficiency. Hence, sustainable management strategies are urgently needed to balance yield, enhance nutrient efficiency, and conserve soil nitrogen throughout the annual cycle.

**Methods:**

This 3-year field experiment investigated an innovative management program’s effects on yield, biomass, green leaf area index, nutrient dynamics, soil residual nutrients, and environmental cost in a wheat‒maize rotation. Two distinct agronomic technology integration management treatments were included, namely, (1) soil–crop management according to farmers’ practices (FP) and (2) an integrated knowledge and innovative management program (IMP). The experiments were conducted over two growing seasons (winter wheat and summer maize) to capture interannual variability and system-level responses.

**Results:**

Compared with the FP treatment, the IMP treatment significantly increased the grain yield, nitrogen use efficiency, and phosphorus use efficiency by 12.7%, 47.6%, and 47.7%, respectively, and decreased reactive nitrogen losses, greenhouse gas emissions, and nitrogen and carbon footprints by 29.1%, 11.0%, 38.1%, and 20.5%, respectively. Random forest modeling identified that IMP enhances yield through increased green leaf area index and improved N and P use efficiency, where enhanced uptake drives higher utilization efficiency. Village-level scenario analysis indicated that the IMP treatment increased yield and economic benefits while reducing nutrient inputs.

**Discussion:**

This study demonstrates that the IMP treatment can achieve both high productivity and low environmental impacts in intensive wheat‒maize systems in the North China Plain, offering actionable guidelines for sustainable smallholder farming and regional agricultural management.

## 1 Introduction

Agriculture faces the dual challenge of increasing yields while decreasing environmental costs (Chen et al., 2014; Wang et al., 2021). Wheat and maize represent the world’s top nitrogen (N)-consuming crops. Their sustainable production is particularly crucial for ensuring food security (Wang et al., 2024a). Wheat–maize rotation is a dominant double-cropping system in the North China Plain (NCP) that relies on a high-input fertilizer management model, which results in soil degradation and limited yield gains (Cao et al., 2024; Wang et al., 2023b). The primary factors limiting crop yields are soil fertility management and agronomic practices, not natural climatic potential (Wang et al., 2023a). Inefficient input utilization and a mismatch between nutrient supply and crop demand constitute major challenges to sustaining crop yields and soil fertility over the long term (Zhou et al., 2022). More than 50% of global N fertilizer inputs are not utilized because of excessive application (Tilman et al., 2002). Moreover, mismatches between phosphorus (P) fertilizer types, crops, and soil conditions lead to significant P waste (Liang et al., 2024). Although soil potassium (K) reserves are abundant, 90–98% of this K pool is present in mineral-bound forms that are difficult for plants to absorb, resulting in K balance deficits in numerous agricultural systems (Zörb et al., 2014; Niu et al., 2013; Zhao et al., 2014; Qaswar et al., 2020). Excessive fertilization can lead to reduced yields, low nutrient use efficiency, nutrient imbalances, soil structure degradation, and a decrease in soil productivity (Bei et al., 2018; Han et al., 2015; Zhang et al., 2018). Chronically excessive application of N impedes its remobilization from vegetative organs to grains, leading to a long-term decrease in the N harvest index (Hasanain et al., 2025). Early wheat sowing risks frost damage because of premature growth, whereas delayed sowing inhibits tillering and ear development (Liu et al., 2025a). An excessively low maize planting density directly results in a reduced number of ears harvested (Liu et al., 2025a). The extensive application of fertilizers and use of agricultural machinery lead to excessive greenhouse gas (GHG) emissions (Hasanain et al., 2025). Therefore, developing new technologies to increase yields and reduce environmental costs in wheat/maize rotation systems is essential for ensuring sustainable agricultural development.

Agriculture is a primary source of GHG emissions, particularly CO_2_, posing a serious threat to global climate and sustainable development (Li et al., 2023). China, as a leading developing country, proposed “dual-carbon (C)” goals in 2020 (Yang et al., 2022). However, global agriculture and forestry account for 23% of total anthropogenic GHG emissions, highlighting the critical role of agriculture in global mitigation efforts (Wang et al., 2023c). In 2020, China accounted for approximately 30.7% of global C emissions (Zhang et al., 2022). Moreover, croplands act as both C sinks and sources in the C cycle (Yan et al., 2021). Under these circumstances, reconciling agricultural emission reduction with crop yield maintenance has become a critical challenge. Existing agricultural practices are constrained in their dual goals of increasing yields and reducing environmental costs through inadequate technology integration (Paramesh et al., 2023). In response to these constraints, Chen et al. (2011) established an integrated soil–crop system management (ISSM) framework. The use of the ISSM approach increased grain yield and N use efficiency (NUE) in maize and wheat and reduced reactive N loss (RNL) and GHG emissions, as demonstrated in trials at 153 sites across the major agricultural ecoregions of China (Chen et al., 2014; Wang et al., 2024b, 2025). Subsequently, Zhang et al. (2024a) implemented the ISSM framework under ridge–furrow planting with full-film mulching, demonstrating that the integration of high-density planting and optimized fertilization could sustain both high yield and soil fertility in semiarid farmlands. Wang et al. (2021) subsequently proposed the integrated knowledge and product strategy (IKPS), in which ISSM practices are combined with products to ensure food and nutritional security while reducing environmental impacts on vegetables. However, vegetable and wheat–maize rotation systems differ fundamentally in terms of fertilizer input intensity, nutrient demand dynamics, and environmental cost profiles (Deng et al., 2023; Wang et al., 2019). Therefore, when the IKPS was transferred to the wheat–maize rotation system, priority was given to cultivation and nutrient optimization. Water management was excluded because of regional heterogeneity (rainfall, irrigation infrastructure, and policy), and farming management was excluded because of its long-term nature. Both aspects are reserved for future research to further refine the framework. In addition, the existing technical strategies for integrating knowledge and fertilizer products in the wheat–maize rotation system remain unclear.

Farmers with low yields and low nutrient utilization efficiency account for a high proportion of the total number of farmers on the NCP. There is much room for improvement in yield and nutrient utilization efficiency (Chen et al., 2018). The results of an analysis of 241 farm plots in the NCP via the Decision Support System of Agrotechnology Transfer (DSSAT)–Crop Environment Resource Synthesis (CERES) model revealed that synergistic high yields with efficient N use were achieved in only 15% of winter wheat fields and 4% of summer maize fields (Li et al., 2019). The zero-distance + participatory model of the Science and Technology Backyard (STB) not only increased technology adoption rates from 17.9% to 53.5% but also encouraged smallholders to increase grain yields by more than 30% and to reduce N emissions by half, thereby creating scalable models for efficient agriculture (Chen et al., 2014; Zhang et al., 2016). In this study, an innovative management program of integrated knowledge and product management (IMP) was developed for a wheat/maize rotation system. This study aimed to examine (1) the ability of the proposed IMP to increase yields and reduce the environmental impacts of the wheat/maize rotation system and (2) the role of the IMP in increasing production and decreasing emissions of smallholder farmers in a village scenario combined with the STB.

## 2 Materials and methods

### 2.1 Study area

Experiments were conducted from 2020 to 2022 in Laoling city (37°42′ N, 117°09′ E), Shandong Province, China. This region experiences warm temperate, continental, subhumid, and monsoonal climatic conditions. The region experiences an average annual rainfall of 694.4 mm, which is concentrated mainly from June to September. The average annual temperature is 14.1 °C (**Supplementary Figure S1**). Winter wheat–summer maize rotation constitutes a typical rotation system in this area. The winter wheat growing season extends from early October to early June. The summer maize growing season ranges from early June to early October. The characteristics of the initial topsoil layer (0–20 cm) are as follows: a pH of 8.6, an organic matter content of 23.1 g kg^-1^, a hydrolyzed N content of 93.6 mg kg^-1^, an available P content of 12.4 mg kg^-1^, and an available K content of 135.5 g kg^-1^.

### 2.2 Experimental design

Field trials were conducted from 2020–2022. The trials included two treatments and four replicates in a randomized complete block design. The two treatments were (1) soil–crop management according to farmers’ practices (FP) and (2) the proposed IMP treatment. The length and width of each plot were 15 and 5 m, respectively. The wheat cultivars were LX77 and JM22, and the maize cultivars were ZD958 and DH605. Winter wheat top-dressing was applied at the regreening stage in FP and at the jointing stage in IMP, with all P and K fertilizers used as basal treatments. Summer maize received topdressing at flare opening and chemical control at V8. Foliar ZnSO_4_·7H_2_O (0.4%, 800 L·ha^-1^) was sprayed at jointing and heading stages for winter wheat and at the jointing and twelfth leaf stages for summer maize, ensuring leaf wetting without runoff. Compared with the FP treatments, the IMP treatment reduced the seeding rate, planting density, and N application rate but increased the P fertilizer rate and the proportion of topdressing N during the wheat season. During the maize season, the IMP treatment decreased the N and P rates and increased the plant density and K rate (**Table 1**). Winter wheat was planted on October 19, 2020, and 27 October 2021 and harvested on June 15, 2021, and 18 June 2022. Summer maize was planted on June 18, 2021, and June 25, 2022, and harvested on October 13, 2021, and October 6, 2022, in the FP treatment and seven days later in the IMP treatment. Artificial weeding and other plant protection measures were implemented as needed to control weeds, insects, and diseases. All the straw was returned to the field without tillage treatment after the wheat was harvested. In contrast, straw was not returned to the field after the maize was harvested. The field was rotated twice. Notably, this study focused on cultivation and nutrient optimization but did not consider water management. Given the importance of water–nitrogen synergy for crop performance, future work should integrate water management to further increase the sustainability and productivity of rotation systems.

**Table 1:** Nutrient input of winter wheat/summer maize.

Crops	Treatment	Variety	Seeding Rate (kg ha^-1^)/Planting density (plants ha^-1^)	Fertilizer application rate (kg ha^-1^)				Micronutrients fertilizer
				N		P_2_O_5_	K_2_O	
				Total inputs	Basal and topdressing fertilizer ratio			
Wheat	FP	LX77	285	284	4:6	134	40	–
	IMP	JM22	225	210	3:7	100	60	Foliar application of zinc fertilizer
Maize	FP	ZD958	6.3	269	–	65	40	–
	IMP	DH605	7.5	210	1:2	50	60	Foliar application of zinc fertilizer; Chemical regulator

### 2.3 Sampling and measurement

Wheat plants were sampled at five developmental stages, namely, at the regreening stage (RS), jointing stage (JS), heading stage (HS), filling stage (FS), and maturity stage (MS). Maize plants were sampled at the following five developmental stages: the sixth leaf stage (V6), twelfth leaf stage (V12), silking stage (R1), filling stage (R3), and maturity stage (R6). At the five growth stages of wheat and maize, four representative wheat plants and three representative maize plants per plot were randomly selected, and the green leaf length and width values were measured. The leaf area (LA) per plant was calculated as the leaf length times the maximum leaf width using coefficients of 0.83 for wheat and 0.75 for maize. The green leaf area index (GLAI) was calculated as the LA multiplied by the number of plants per unit area. At each growth stage, ten wheat plants and three maize plants were randomly selected from each plot. The samples were partitioned into leaves and stems prior to the silking stage, while the postsilking samples were separated into leaves, stems, and grains. All the plant samples were heated at 105 °C for 30 min, dried at 70 °C to a constant weight and weighed to obtain dry matter. The dried plant samples were subsequently ground. The samples were boiled with H_2_SO_4_–H_2_O_2_. The total N content was determined via the Kjeldahl N determination method (Bao, 2000). The total P content was determined with the vanadium–molybdenum–yellow colorimetric method (Bao, 2000). The total K content was determined through flame spectrophotometry (Bao, 2000). The following indicators were calculated for both N and P:


NupE(PupE)=total plant N(Puptake)/N(P)input

NutE(PutE)=grain yield/total plant N(P)uptake

NUE(PUE)=grain yield/N(P)input

NHI(PHI)=[grain N(P)accumulation/total aboveground N(P)uptake]×100%

Among them, *NupE (PupE)* denotes N and P uptake efficiency, *NutE (PutE)* denotes N and P utilization efficiency, *NuE (PuE)* denotes N and P use efficiency, and *NHI (PHI)* denotes the N and P harvest index.

At the physiological maturity stage, 1 m^2^ (1 × 1 m) of winter wheat was randomly harvested from each plot to determine the grain yield. Twenty consecutive summer maize plants were randomly harvested from each plot to determine the grain yield. The actual row and plant spacing were measured. The seeds were dried in a drying oven at 70 °C to a constant weight. The maize yield was normalized to a standard moisture content of 14%, and the wheat yield was normalized to a standard moisture content of 12.5%. In each plot, soil cores were collected from three depths (0–20, 20–40, and 40–60 cm), and the collected soil samples were homogenized and air dried. The contents of soil alkali-hydrolyzable N, available P, and available K were analyzed via alkali-hydrolysis diffusion, NaHCO_3_–molybdenum–antimony spectrophotometry, and NH_4_OAC–flame photometry methods, respectively (Bao, 2000).

### 2.4 Calculation of environmental and economic benefits

#### 2.4.1 System boundary and functional units

The life cycle assessment method is an internationally recognized method for evaluating the environmental impacts of all links throughout the entire life cycle of a product from cradle to grave (Brentrup et al., 2004; Rebitzer et al., 2004). In this study, the system boundary was defined as the agricultural materials stage (MTS) and the field tillage stage (FTS) of the entire process of winter wheat/summer maize planting. The agricultural materials phase includes the production and transportation of fertilizers, pesticides, and diesel oil. The tillage phase includes field application of N fertilizer and other management practices during planting (such as irrigation, pesticide application, and mechanical equipment use).

#### 2.4.2 Reactive N losses and N footprint

In this study, we calculated the RNL from winter wheat/summer maize N application, including NH_3_ volatilization, NO_3_^-^ leaching, and N_2_O emission, as follows (Cui et al., 2018).

For winter wheat, the following applies:


$${\text{NH}}_{3}\;volatilization=2.69+0.069\times {\text{N}}_{\text{rate}}$$

$${\text{N}}_{2}O\;emission=0.50\times {\text{e}}^{\left({0.0032\times \text{N}}_{\text{rate}}\right)}$$

$${\text{NO}}_{3}^{-}\;leaching=3.63\times {\text{e}}^{\left({0.0080\times \text{N}}_{\text{rate}}\right)}$$

For summer maize, the following applies:


$${\text{NH}}_{3}\;volatilization=7.98+0.099\times {\text{N}}_{\text{rate}}$$

$${\text{N}}_{2}O\;emission=0.99\times {\text{e}}^{\left({0.0047\times \text{N}}_{\text{rate}}\right)}$$

$${\text{NO}}_{3}^{-}\;leaching=10.7\times {\text{e}}^{\left({0.0060\times \text{N}}_{\text{rate}}\right)}$$

where *N_rate_* denotes the amount of N fertilizer applied (kg N ha^-1^). In this study, the N footprint (kg N Mg^-1^) was defined as the amount of RNL per unit yield.

#### 2.4.3 GHG emissions and C footprint

In this study, MTS and FTS denote the agricultural materials stage and the field tillage stage, respectively, and GHG emissions (kg CO_2_ eq ha^-1^) refer to the total GHG emissions per unit cultivated land area.


$$GHG=\left({\text{GHG}}_{\text{m}}+{\text{GHG}}_{\text{t}}\right)\times {\text{N}}_{\text{fert}}+{\text{N}}_{{\text{N}}_{2}\text{O}}\times 44/28\times 298+{\text{GHG}}_{\text{others}}$$

$$Total\;{\text{N}}_{2}O={\text{N}}_{2}O\;diret+1\%\times {\text{NH}}_{3}+0.75\%\times {\text{NO}}_{3}^{-}\;leaching$$

where *GHG_m_* and *GHG_t_* denote the GHG emission factors for N fertilizer production and transportation, respectively. Moreover, *GHG_others_* denotes the GHG emissions from the production and transportation of phosphate and potash fertilizers, pesticides, fungicides, and herbicides; the GHG emissions from the production, transportation, and application of pesticides; and the GHG emissions from the combustion of diesel fuel during crop seeding, harvesting, tillage, and electricity generation for irrigation. In addition, 44/28 is a coefficient for converting N into N_2_O, and 298 is the equivalent coefficient of the GHG effect of N_2_O per unit mass compared with that of CO_2_.

Total N_2_O denotes the total nitrous oxide emissions produced after the field application of N fertilizer, including direct and indirect N_2_O emissions caused by NH_3_ volatilization and NH_3_^-^ leaching. The default values for the indirect emission factors of NH_3_ volatilization and NH_3_^-^ leaching are 1% and 0.75%, respectively (IPCC, 2013).

#### 2.4.4 Economic benefit


Economic benefit=Yield×Grain price-Fertilizer fees-Seed costs-Machinery expenses-Pesticide cost-Irrigation cost

where *Grain price* (CNY kg^-1^) denotes the local grain price; *Fertilizer fees* (CNY ha^-1^) denote the input cost of fertilizers; *Seed costs* (CNY ha^-1^) denote the price of seeds; *Machinery expenses* (CNY ha^-1^) denote the mechanical cost of land preparation, sowing, and harvesting; *Pesticide cost* (CNY ha^-1^) denotes the input of pesticides for crop cultivation; and *Irrigation cost* (CNY ha^-1^) denotes the electricity cost for irrigation.

### 2.5 Field survey

A field experiment was conducted in Laoling city from 2020 to 2022 to obtain parameters under the IMP treatment. Concurrently, a farmer survey was conducted using a point to village scale extrapolation method, and the survey data were used to represent the FP treatment. We used a structured questionnaire to ensure data consistency and reduce ambiguity. The field survey was conducted in four representative townships with a wheat–maize rotation in Laoling city (Guojia, Zhuji, Yangan, and Hualou). In each township, four villages were randomly selected, and 10–15 households were randomly surveyed per village. After questionnaires with missing data were excluded and outliers were removed via logical checks, 156 valid records were retained for analysis. The survey covered three categories of information: (1) cultivation characteristics (planting area, sowing date, harvest date, and yield); (2) water, fertilizer, and pesticide management (irrigation amount and timing, fertilizer type, nutrient rate, application method, timing, and frequency); and (3) energy inputs (diesel for land preparation and tillage, electricity for irrigation). Integration of the experimental and survey results enabled the construction of a multi-scale evaluation framework from field to village level, allowing the extrapolation of field-scale performance to actual production conditions and facilitating the assessment of the potential environmental benefits of the IMP treatment. The above survey data provide empirical support for the subsequent assessment of the environmental benefits of IMP at the village scale.

### 2.6 Statistical analysis

All the data were analyzed with SPSS 27.0 software. Statistical normality was assessed using the Shapiro–Wilk test (Souza et al., 2023). On the basis of the test results, between-group differences were analyzed with an independent samples t test for normally distributed data or with the nonparametric Mann–Whitney U test for nonnormally distributed data (Alnaeem et al., 2026). To evaluate the importance of each indicator for the yield, a random forest model was constructed using the randomForest package (v4.7–1.1) in R (v4.3.2). The model was trained with 500 trees and mtry = floor(sqrt(p)), where p is the number of predictors after dummy encoding, standardization, and near-zero variance filtering. Model performance was evaluated using out-of-bag (OOB) internal cross-validation. Variable importance was assessed by %IncMSE, normalized to relative importance (%), and complemented with Pearson correlation coefficients between each predictor and the response variable.

## 3 Results

### 3.1 Yield and growth of winter wheat/summer maize

Compared with that in the FP treatment, the annual yield (wheat and maize) significantly increased by 12.7% on average in the IMP treatment (**Figure 1a**). During the two growing seasons, compared with those in the FP treatment, the winter wheat yields in the IMP treatment increased by 13.1% and 12.4% (**Figure 1a**). The increase in winter wheat yield under the IMP treatment in 2020–2021 could be attributed mainly to substantial increases in the number of ears, the number of kernels per spike, and the 1000-kernel weight (**Figure 1**). Despite the different seeding rates between the two treatments, there was no significant difference in the number of wheat ears or the number of kernels per spike from 2021–2022 (**Figures 1b, c**). These results can be attributed to the more effective tiller production under the IMP treatment. Additionally, the 1000-grain weight of winter wheat in the IMP treatment was significantly greater than that in the FP treatment from 2021–2022, with an increase of 7.4% (**Figure 1d**).

**Figure 1:**
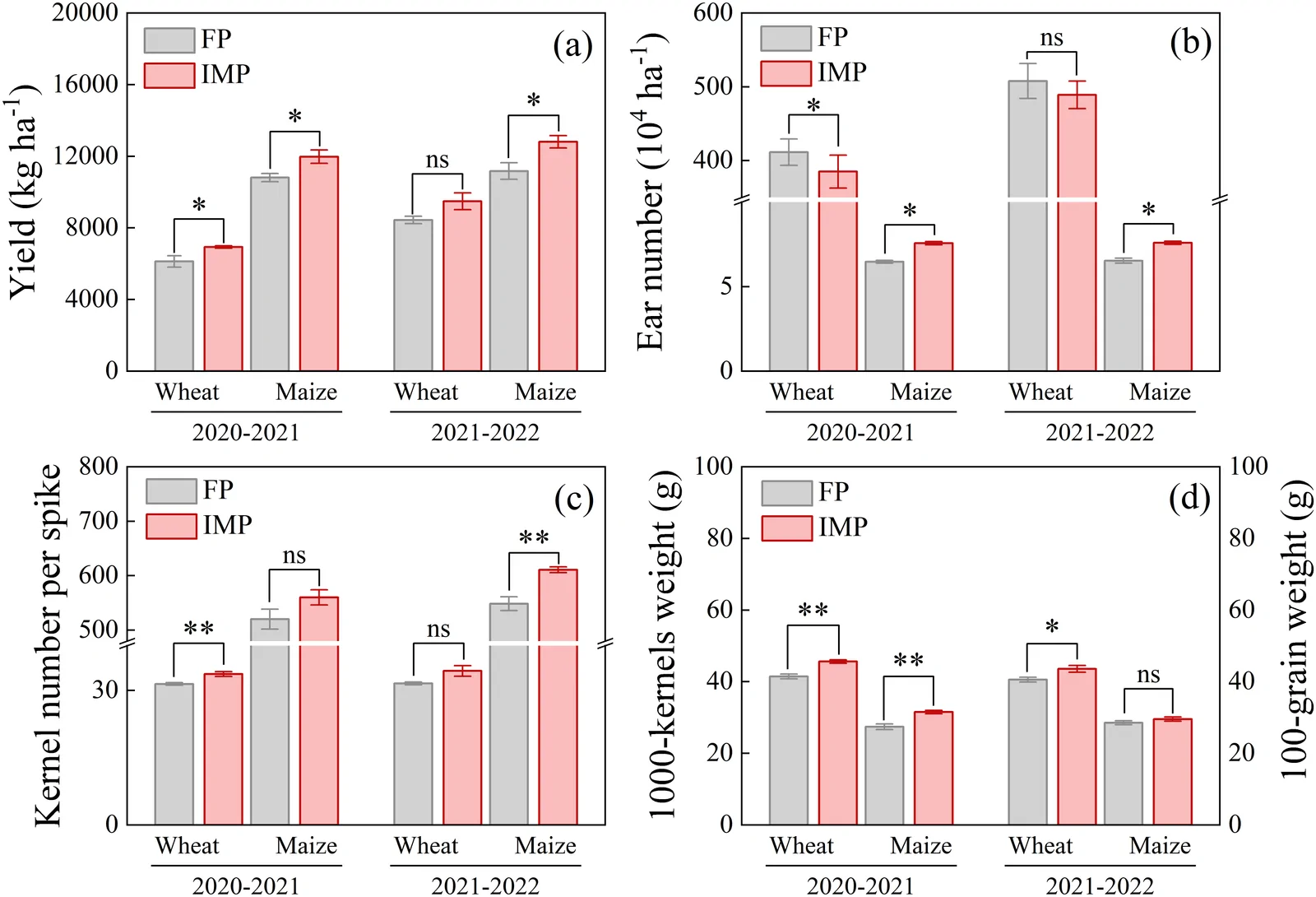
Effects of innovative management program on winter wheat/summer maize yield and their component factors. **(a–d)** represent yield, ear number, kernel number per spike and grain weight. Wheat is 1000-kernels weight, maize is 100-grain weight. FP represents farmer practices, IMP represents innovative management program. ns: not significant (P > 0.05). * Significant at P < 0.05. ** Significant at P < 0.01.

During the two growing seasons, compared with those in the FP treatment, the maize yields in the IMP treatment significantly increased by 10.8% and 14.7% (**Figure 1a**). The number of ears in the IMP treatment was significantly greater than that in the FP treatment during both years, with an average increase of 16.6%, owing to the increased summer maize planting density under the innovative management program design (**Figure 1b**). Compared with that in the FP treatment, the number of kernels per spike in the IMP treatment from 2021–2022 was significantly greater (by 11.4%) (**Figure 1c**). These results can be attributed to the greater number of kernels per spike achieved by the summer maize hybrid DH605 in the IMP treatment. Compared with that in the FP treatment, the 100-grain weight in the IMP treatment was significantly greater (by 14.9%) from 2020–2021 (**Figure 1d**).

### 3.2 Nutrient accumulation, utilization and residue

The GLAI of winter wheat exhibits a unimodal curve throughout its development, which is characterized by an increase to the maximum value at the HS, followed by a steady decrease. In the wheat/maize rotation system, compared with that in the FP treatment, the two-year average GLAI in the IMP treatment increased by 4.3%. During the 2020–2021 growing season, no significant changes were observed in the GLAI of wheat across all growth stages. From 2021–2022, compared with those in the IMP treatment, the GLAI values in the FP treatment significantly increased by 25.4% at the JS and by 19.3% at the FS. In addition, at the MS, yield was 38.9% greater under the IMP treatment than under the FP treatment (P< 0.001). The GLAI of summer maize first increased but then decreased from the V6 stage to the R6 stage. The rate of increase in the GLAI of summer maize was greatest from the V6 stage to the V12 stage and reached its maximum value at the R1 stage. Compared with those in the FP treatment, the GLAI values in the IMP treatment increased by 11.3% (R3) and 24.5% (R6) from 2020–2021 and by 25.9%, 8.0%, and 6.9% from the V12 stage to the R3 stage from 2021–2022, respectively, with all the differences being significant (**Figure 2**).

**Figure 2:**
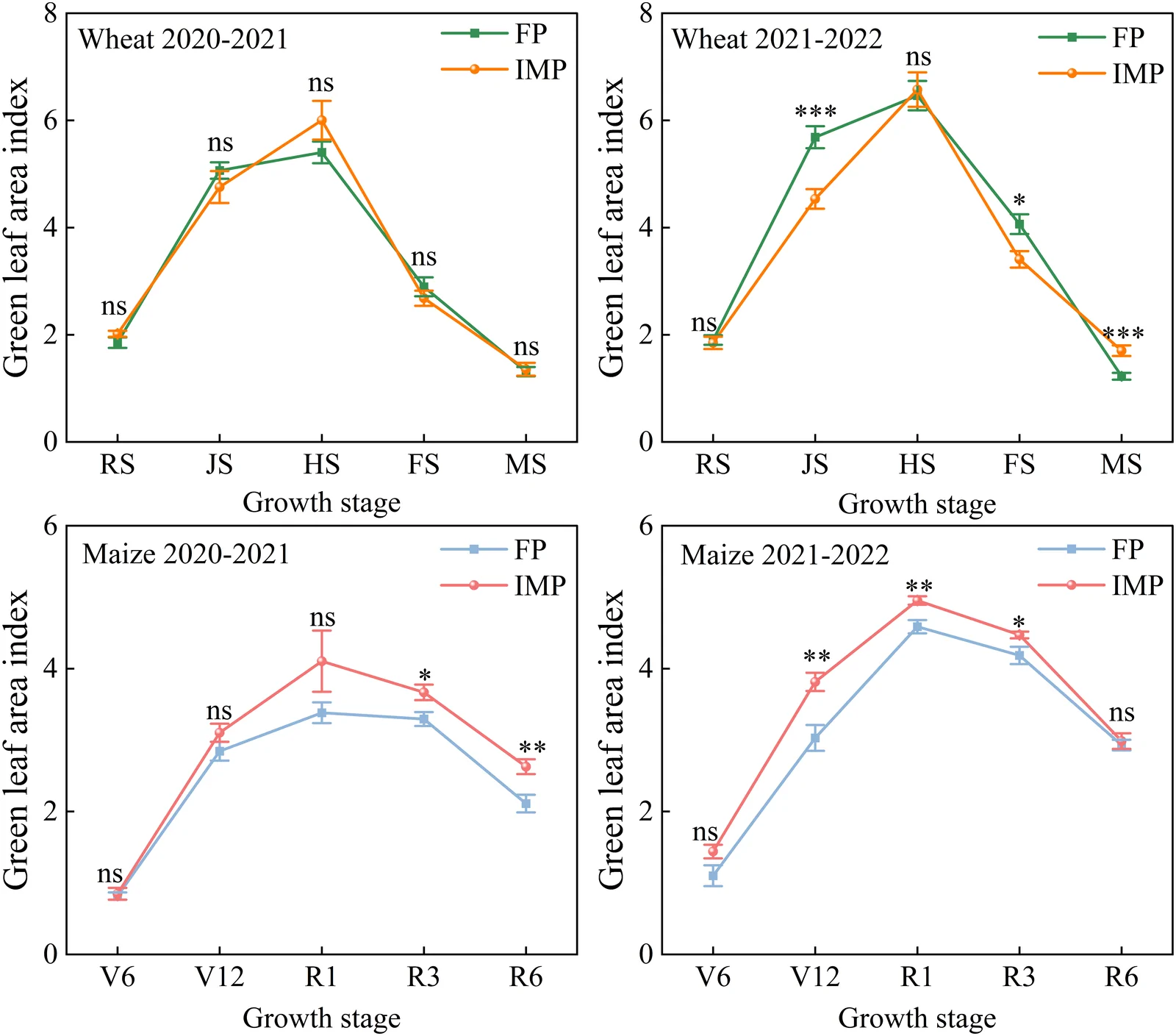
Effects of innovative management program on green leaf area index of winter wheat/summer maize in 2020–2021 and 2021**–**2022. RS, JS, HS, FS, and MS represent wheat’s regreening stage, jointing stage, heading stage, filling stage, and maturity stage. V6, V12, R1, R3, and R6 represent maize’s sixth leaf stage, twelfth leaf stage, silking stage, filling stage and maturity stage. FP represents farmer practices; IMP represents innovative management program. ns: not significant (P > 0.05). * Significant at P< 0.05. ** Significant at P< 0.01. *** Significant at P< 0.001.

At the maturity stage of winter wheat and summer maize, grains accounted for the greatest proportion of dry matter accumulation in summer maize, followed by stems and leaves. In the wheat/maize rotation system, compared with that in the FP treatment, the two-year average dry matter accumulation level in the IMP treatment increased by 5.8% (**Figure 3**). During the MS stage of winter wheat from 2020–2021, the grain dry matter accumulation was 14.7% greater in the IMP treatment than in the FP treatment (**Figure 3a**). From 2020–2021, the level of dry matter accumulation in summer maize was significantly greater in the IMP treatment than in the FP treatment, with increases of 39.7% and 13.0% in stems at the V6 and R3 stages, respectively, 17.5% in leaves at the R6 stage, and 38.3% and 10.8% in grains at the R3 and R6 stages, respectively. From 2021–2022, the level of dry matter accumulation was also significantly greater in the IMP treatment than in the FP treatment, with increases of 17.1% in stems at the R1 stage, 47.4% and 14.6% in leaves at the V12 and R1 stages, respectively, and 14.7% in grains at the R6 stage. The level of whole-plant dry matter accumulation was significantly greater in the IMP treatment than in the FP treatment at the R3 to R6 stages from 2020–2021 (by 24.9% and 12.9%, respectively) and at the V6 to V12 stages from 2021–2022 (by 25.8% and 22.5%, respectively) (**Figure 3b**).

**Figure 3:**
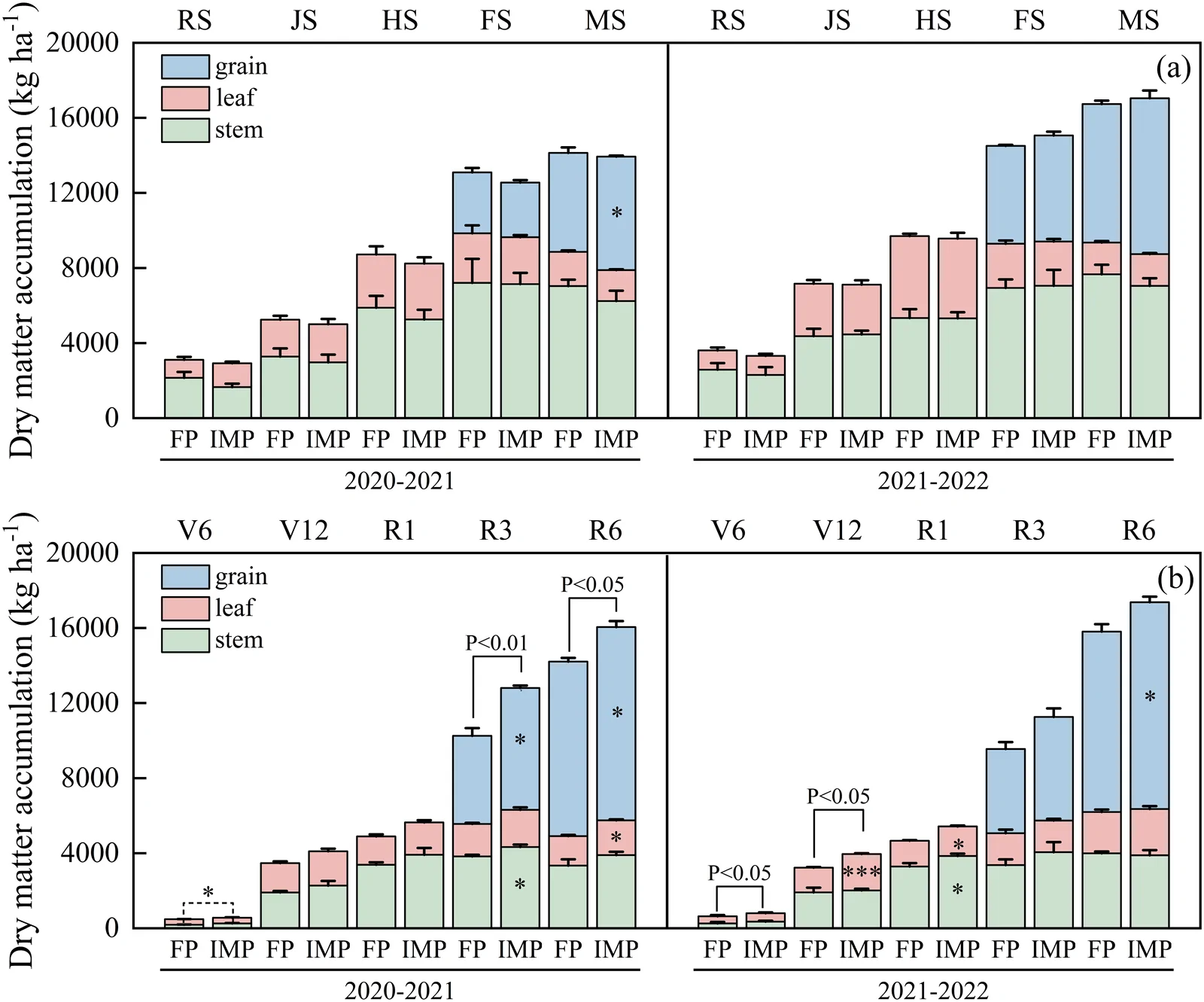
Effects of innovative management program on dry matter accumulation of winter wheat/summer maize in 2020–2021 and 2021**–**2022. **(a, b)** represent winter wheat and summer maize, respectively. RS, JS, HS, FS, and MS represent wheat’s regreening stage, jointing stage, heading stage, filling stage, and maturity stage. V6, V12, R1, R3, and R6 represent maize’s sixth leaf stage, twelfth leaf stage, silking stage, filling stage, maturity stage. FP represents farmer practices; IMP represents Innovative management program. ns: not significant (P > 0.05). * Significant at P< 0.05. ** Significant at P< 0.01. *** Significant at P< 0.001. * indicates significant differences among organs; P value represents significant differences among whole-plant.

In the wheat/maize rotation system, compared with those in the FP treatment, the two-year average N and P accumulation in the IMP treatment increased by 8.4% and 19.5%, respectively (**Figures 4**, **5**). There were no significant differences in N accumulation among the treatments during the two wheat seasons. In summer maize, the level of N accumulation in the IMP treatment was significantly greater than that in the FP treatment by 11.6–49.0% at the V12 to R6 stages from 2020–2021 and by 26.3–34.8% at the R1 to R3 stages from 2021–2022 (**Figure 4**). From 2020–2021, there was no significant difference in P accumulation in wheat. However, the level of P accumulation in the IMP treatment was significantly greater (15.8%) than that in the FP treatment at the MS stage from 2021–2022. From 2020–2021, at the V12 to R6 stages (especially at the R3 stage), the level of P accumulation in maize plants was significantly greater in the IMP treatment than in the FP treatment, with increases of 30.7–68.4%. The IMP treatment resulted in a significant increase of 20.3–60.8% across all the maize growth stages from 2021–2022 (**Figure 5**).

**Figure 4:**
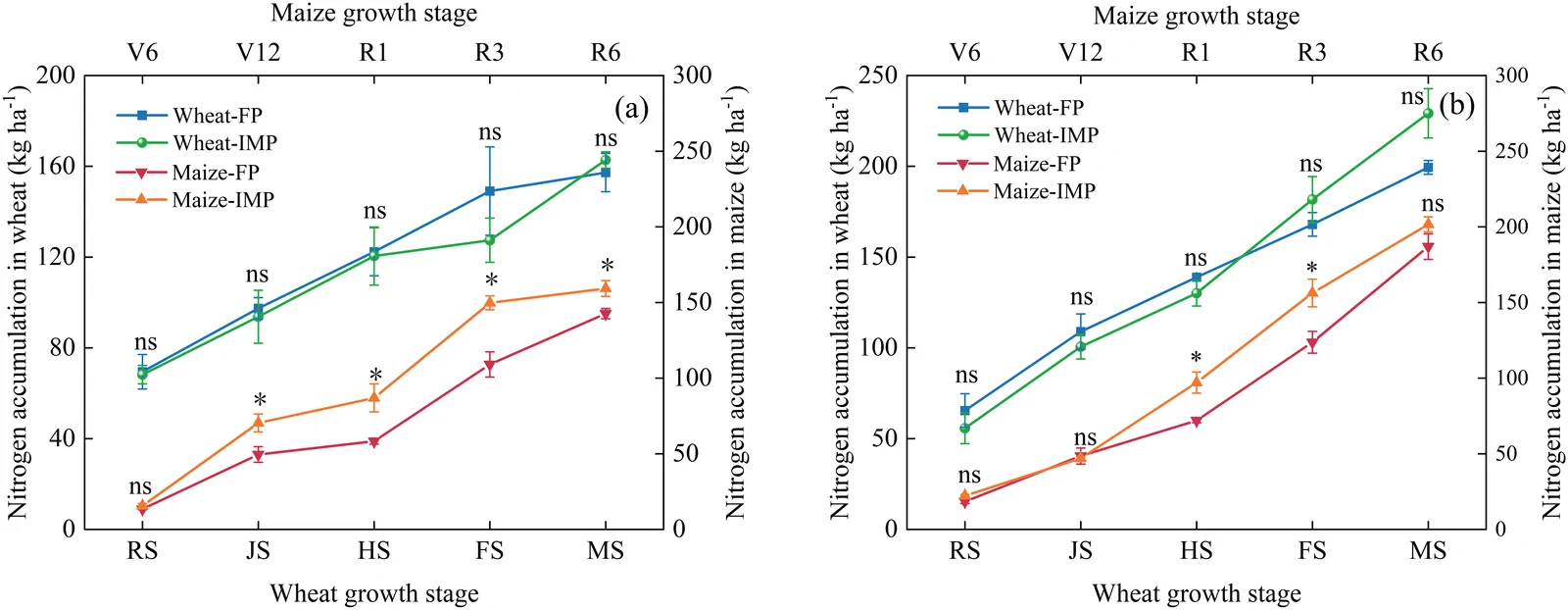
Effects of innovative management program on N accumulation in winter wheat/summer maize in 2020–2021 and 2021–2022. **(a, b)** represent winter wheat and summer maize, respectively. RS, JS, HS, FS, and MS represent wheat’s regreening stage, jointing stage, heading stage, filling stage, and maturity stage. V6, V12, R1, R3, and R6 represent maize’s sixth leaf stage, twelfth leaf stage, silking stage, filling stage and maturity stage. FP represents farmer practices; IMP represents Innovative management program. ns: not significant (P > 0.05). * Significant at P< 0.05.

**Figure 5:**
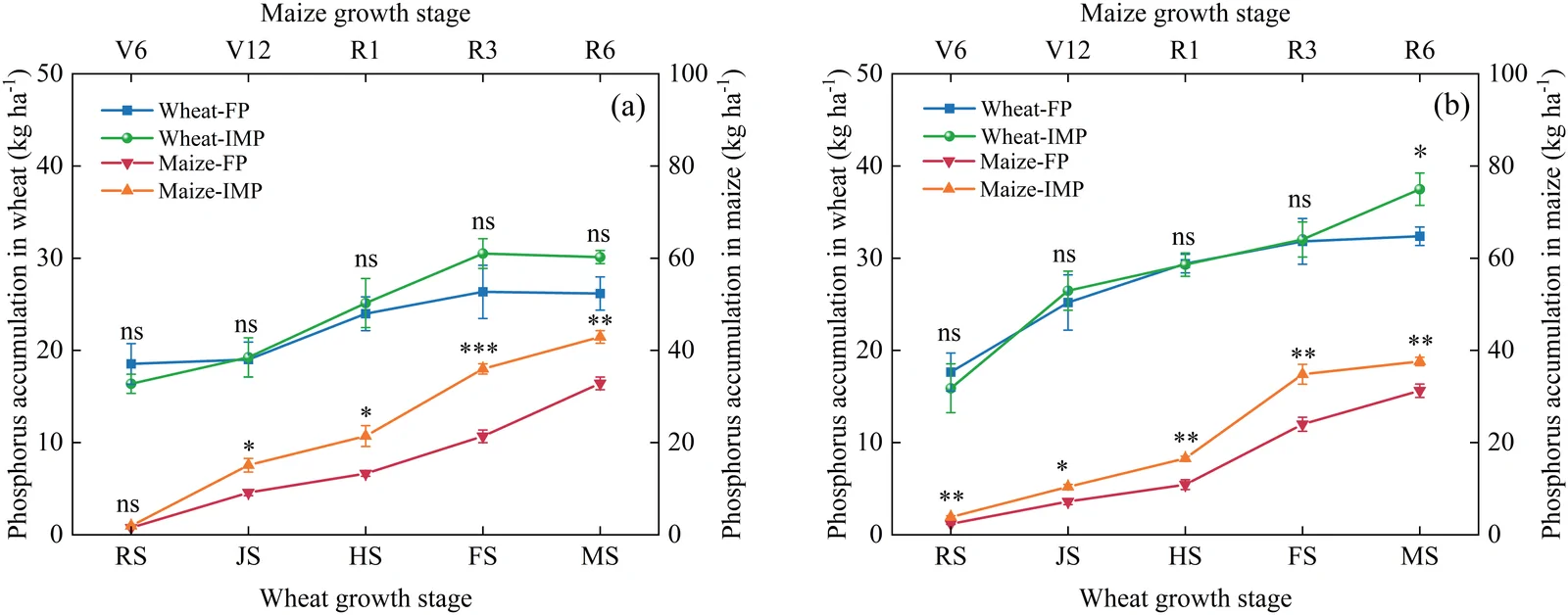
Effects of innovative management program on P accumulation in winter wheat/summer maize in 2020–2021 and 2021–2022. **(a, b)** represent winter wheat and summer maize, respectively. RS, JS, HS, FS, and MS represent wheat’s regreening stage, jointing stage, heading stage, filling stage, and maturity stage. V6, V12, R1, R3, and R6 represent maize’s sixth leaf stage, twelfth leaf stage, silking stage, filling stage, maturity stage. FP represents farmer practices; IMP represents Innovative management program. FP represents farmer practices; IMP represents innovative management program. ns: not significant (P > 0.05). * Significant at P< 0.05. ** Significant at P< 0.01. *** Significant at P< 0.001.

In the wheat/maize rotation system, the IMP treatment significantly increased the N and P use efficiency, increasing NupE, NUE, PupE, and PUE by 44.5%, 47.6%, 48.2%, and 47.7%, respectively (**Tables 2**, **3**). With respect to winter wheat, the IMP treatment significantly increased the NupE, NutE, NUE, NHI, PupE, and PUE by 40.0%, 9.3%, 53.1%, 6.5%, 54.2%, and 51.7%, respectively, from 2020–2021 and increased the NupE, NUE, PupE, and PUE by 55.4%, 52.1%, 55.1%, and 50.7%, respectively, from 2021–2022 (**Table 2**). In summer maize, the IMP treatment significantly increased the NupE, NUE, PupE, and PUE by 43.0%, 41.9%, 45.1%, and 44.0%, respectively, from 2020–2021 and by 38.1%, 46.9%, 56.0%, and 49.0%, respectively, from 2021–2022 (**Table 3**).

**Table 2:** Effects of the innovative management program on N and P use at maturity in winter wheat.

Year	2021				2022			
Treatment	FP	IMP	*t*	*p*	FP	IMP	*t/Z*	*p*
NupE (kg kg^-1^)	0.55 ± 0.03	0.78 ± 0.02	-6.461	<0.001^***^	0.70 ± 0.01	1.09 ± 0.06	-5.888	0.001^**^
NutE (kg kg^-1^)	34.03 ± 0.63	37.19 ± 0.50	-3.943	0.008^**^	37.03 ± 0.54	36.32 ± 1.06	0.594	0.574^ns^
NUE (kg kg^-1^)	18.81 ± 0.86	28.80 ± 0.28	-11.048	<0.001^***^	26.00 ± 0.63	39.54 ± 1.96	-6.572	<0.001^***^
NHI (%)	66.29 ± 1.06	70.63 ± 0.84	-3.215	0.018^*^	71.92 ± 0.2	73.34 ± 1.56	-0.904	0.401^ns^
PupE (kg kg^-1^)	0.20 ± 0.01	0.30 ± 0.01	-6.957	<0.001^***^	0.24 ± 0.01	0.37 ± 0.02	-2.309	0.029^*^
PutE (kg kg^-1^)	205.20 ± 5.55	201.12 ± 4.75	0.559	0.597^ns^	228.26 ± 2.35	221.52 ± 3.40	1.630	0.154^ns^
PUE (kg kg^-1^)	39.87 ± 1.82	60.49 ± 0.58	-10.766	<0.001^***^	55.11 ± 1.34	83.04 ± 4.12	-6.450	<0.001^***^
PHI (%)	73.02 ± 1.56	75.23 ± 1.61	-0.987	0.362^ns^	85.28 ± 1.01	84.33 ± 0.62	0.806	0.451^ns^

ns: not significant (P > 0.05). * Significant at P< 0.05. ** Significant at P<0.01. *** Significant at P< 0.001. Values are means ± SE. Due to non-normal distribution of PupE, the Mann-Whitney U test was applied.

**Table 3:** Effects of the innovative management program on N and P use at maturity in summer maize.

Year	2021				2022			
Treatment	FP	IMP	*t*	*p*	FP	IMP	*t*	*p*
NupE (kg kg^-1^)	0.53 ± 0.01	0.76 ± 0.02	-8.161	<0.001^***^	0.70 ± 0.03	0.96 ± 0.02	-6.754	<0.001^***^
NutE (kg kg^-1^)	65.25 ± 1.58	64.71 ± 0.95	0.292	0.780^ns^	51.46 ± 0.9	54.67 ± 1.04	-2.331	0.059^ns^
NUE (kg kg^-1^)	34.57 ± 0.73	49.05 ± 1.53	-8.558	<0.001^***^	35.73 ± 1.47	52.47 ± 1.41	-8.237	<0.001^***^
NHI (%)	72.73 ± 1.25	73.35 ± 1.03	-0.383	0.715^ns^	69.42 ± 1.14	70.17 ± 1.08	-0.474	0.652^ns^
PupE (kg kg^-1^)	2.19 ± 0.05	3.19 ± 0.10	-8.467	<0.001^***^	0.48 ± 0.02	0.75 ± 0.02	-8.879	<0.001^***^
PutE (kg kg^-1^)	284.16 ± 10.15	238.87 ± 1.58	4.410	0.005^**^	307.09 ± 11.85	292.58 ± 2.55	1.198	0.310^ns^
PUE (kg kg^-1^)	143.05 ± 3.01	206.01 ± 6.42	-8.881	<0.001^***^	147.85 ± 6.07	220.36 ± 5.91	-8.560	<0.001^***^
PHI (%)	82.11 ± 0.64	81.97 ± 0.88	0.124	0.905^ns^	82.27 ± 1.09	82.42 ± 0.44	-0.127	0.903^ns^

ns, not significant (P > 0.05). * Significant at P< 0.05. ** Significant at P<0.01. *** Significant at P< 0.001. Values are means ± SE.

No significant difference in the content of soil alkaline-hydrolyzable N was detected across treatments over the two-year wheat/maize rotation period. From 2020–2021, compared with the FP treatment, the IMP treatment significantly increased the available P content in the 0–20 cm soil layer for wheat by 44.3% but significantly decreased it in the 20–40 cm soil layer for maize by 33.3%. From 2021–2022, compared with the FP treatment, the IMP treatment increased the available P content in the 20–40 cm soil layer for wheat by 25.5%, but no significant difference was observed for maize. In terms of the available K content, a significant decrease of 5.4% in the IMP treatment compared with that in the FP treatment was observed only in the 20–40 cm soil layer for maize from 2020–2021 (**Figure 6**).

**Figure 6:**
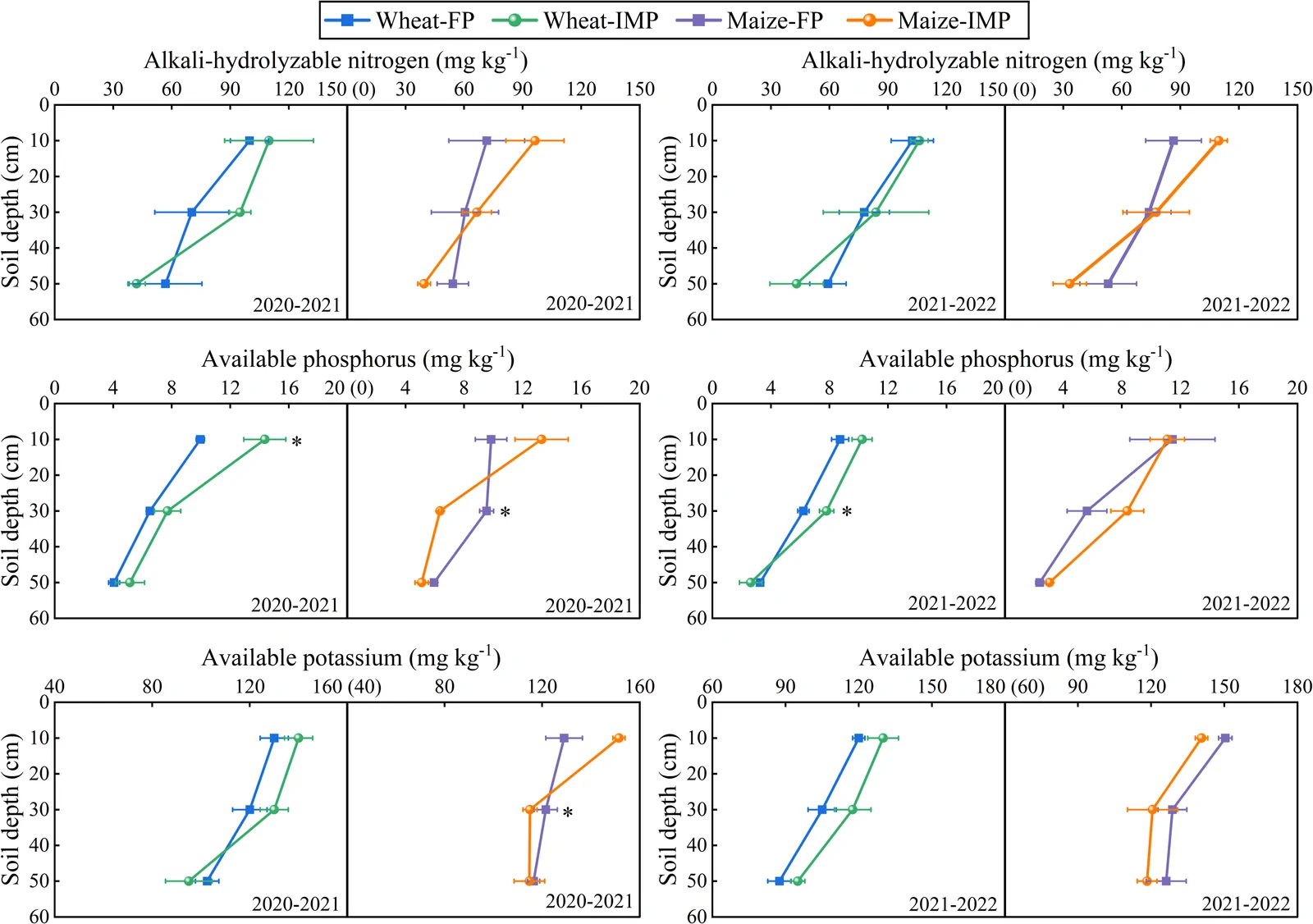
Effects of innovative management program on soil alkali-hydrolyzable N, available P, and available K in winter wheat/summer maize in 2020–2021 and 2021**–**2022. FP represents farmer practices; IMP represents innovative management program. * Significant at P< 0.05.

### 3.3 Environmental costs and economic benefit

In the wheat/maize rotation system, compared with those in the FP treatment, the two-year average RNL and GHG emissions in the IMP treatment decreased by 29.1% and 11.0%, respectively (**Figures 7a, b**). The IMP treatment reduced N_2_O emissions, NH_3_ volatilization, and NO_3_^-^ leaching by 23.4%, 19.2%, and 35.7%, respectively (**Figure 7a**). The IMP treatment decreased the GHG emissions from the MTS-N, FTS-N, and FTS-P fertilizers by 24.4%, 24.1%, and 24.6%, respectively. Irrigation electricity was the main contributor to GHG emissions, followed by FTS-N and MTS-N fertilizers (**Figure 7b**). During the two wheat growing seasons, the IMP treatment reduced RNL and GHG emissions by 35.9% and 8.5%, respectively. The IMP treatment reduced N2O emissions, NH_3_ volatilization, and NO_3_^-^ leaching by 21.1%, 22.9%, and 44.7%, respectively. The IMP treatment decreased the GHG emissions from the MTS-N, FTS-N, and FTS-P fertilizers by 24.9%, 26.1%, and 25.4%, respectively. Irrigation electricity was the main contributor to GHG emissions, followed by FTS-N fertilizers, MTS-N fertilizers, and diesel. During the two maize growing seasons, the IMP treatment reduced RNL and GHG emissions by 24.7% and 14.7%, respectively. The IMP treatment reduced N_2_O emissions, NH3 volatilization, and NO_3_^-^ leaching by 24.2%, 16.9%, and 29.8%, respectively. The IMP treatment decreased the GHG emissions from the MTS-N, FTS-N, and FTS-P fertilizers by 24.2%, 21.9%, and 23.1%, respectively. The main contributor to GHG emissions was FTS-N fertilizers in the summer maize (followed by irrigation electricity and MTS-N fertilizers), but irrigation electricity was the major contributor in the winter wheat (followed by FTS-N and MTS-N fertilizers) (**Figures 7a, b**).

**Figure 7:**
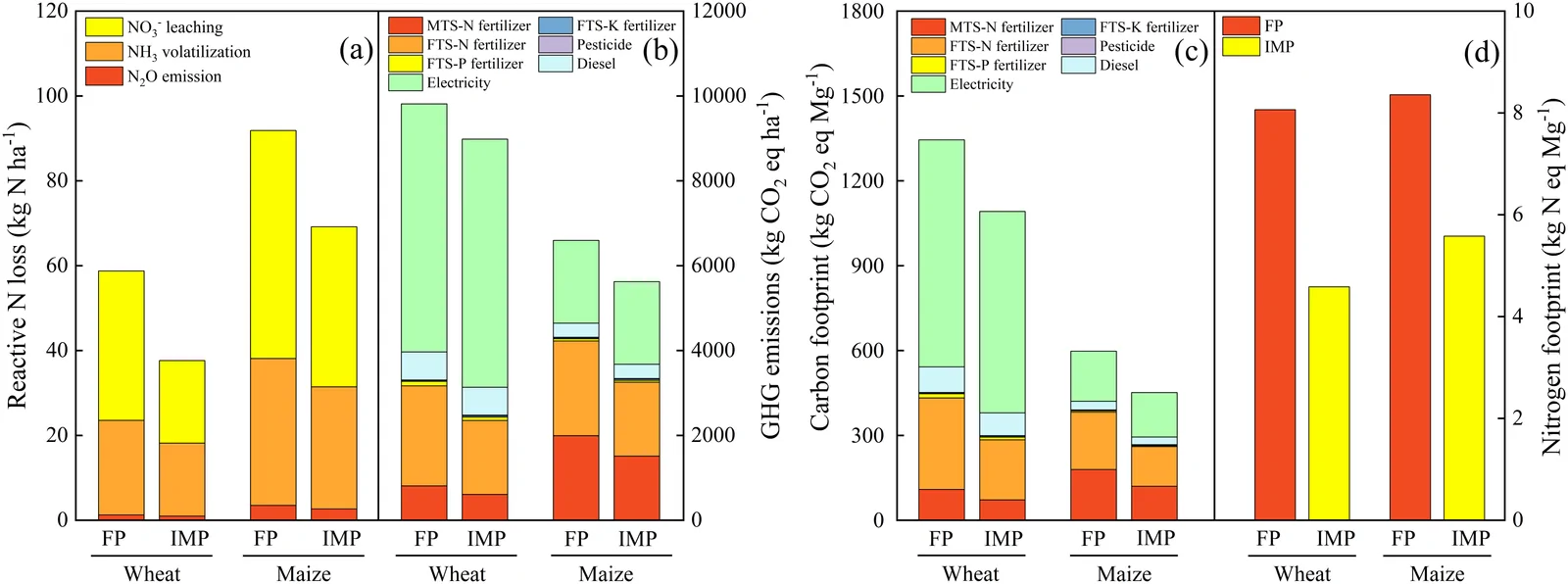
Effects of innovative management program on reactive N loss, greenhouse gas emissions, C footprint and N footprint in winter wheat/summer maize in 2020–2022. **(a–d)** represent RNL, GHG emissions, C footprint and N footprint. FP represents farmer practices; IMP represents innovative management program. MTS: the agricultural materials stage; FTS: the field tillage stage. The data represents a two-year average.

Compared with those in the FP treatment, the two-year average C and N footprints of the wheat/maize rotation system decreased by 20.5% and 38.1%, respectively, in the IMP treatment (**Figures 7c, d**). The C footprint of winter wheat production was greater than that of summer maize. The C footprints of winter wheat and summer maize under the FP treatment were 1347 and 600 kg CO_2_ eq Mg^-1^, respectively, which decreased by 18.8% and 24.4%, respectively, under the IMP treatment. Energy from irrigation was the main contributor to the C footprint of wheat, followed by FTS-N fertilizers. MTS-N fertilizers, FTS-N fertilizers, and irrigation electricity were the primary contributors to the C footprint of maize (**Figure 7c**). The N footprint of summer maize production was greater than that of winter wheat production. The N footprints of winter wheat and summer maize under the FP treatment were 8.1 and 8.4 kg N eq Mg^-1^, respectively, which decreased by 43.1% and 33.2%, respectively, under the IMP treatment (**Figure 7d**).

The IMP treatment increased the total output value of winter wheat/summer maize production and improved the net economic benefits. From 2020–2021, compared with the FP treatment, the IMP treatment increased the total output value by 11.7% to 56635 CNY ha^-1^. Although agricultural input costs rose by 10.1% due to fertilizer optimization, the increase in output far outweighed the additional costs, resulting in a net economic benefit of 46329 CNY ha^-1^–a 12.0% increase. From 2021–2022, compared with the FP treatment, the IMP treatment increased the total output value by 13.7% to 67142 CNY ha^-1^ and the net economic benefits by 14.3%, even as the input costs remained unchanged from those of the previous season (**Supplementary Table S1**).

Overall, the IMP treatment outperformed the FP treatment by increasing the yield and economic benefits by 12.7% and 13.3%, respectively; increasing the N uptake efficiency (NupE) and NUE by 44.5% and 47.6%; and decreasing the RNL, GHG emissions, and N and C footprints by 29.1%, 11.0%, 38.1%, and 20.5%, respectively (**Figure 8**). The random forest model results revealed the importance of individual indicators for yield in the wheat–maize rotation system. The eight indicators with the greatest relative importance were the GLAI (9.1%), PUE (9.0%), NUE (8.6%), NupE (7.9%), PHI (7.9%), number of kernels per ear (7.2%), TN (7.1%), and dry matter (6.0%), all of which were highly significant (P< 0.001). The next most important indicators were NHI (5.2%), TP (4.5%), NutE (4.2%), and TK (3.3%). There were significant differences between years and hybrids (**Figure 9**). A Pearson correlation heatmap revealed that increasing K fertilizer application could increase yield by increasing NUE, PUE, kernel number per ear, and TP content. In contrast, increasing planting density significantly reduced the abovementioned indicators. Additionally, the topdressing ratio was negatively correlated with the GLAI, dry matter accumulation, and TN (**Figure 10**).

**Figure 8:**
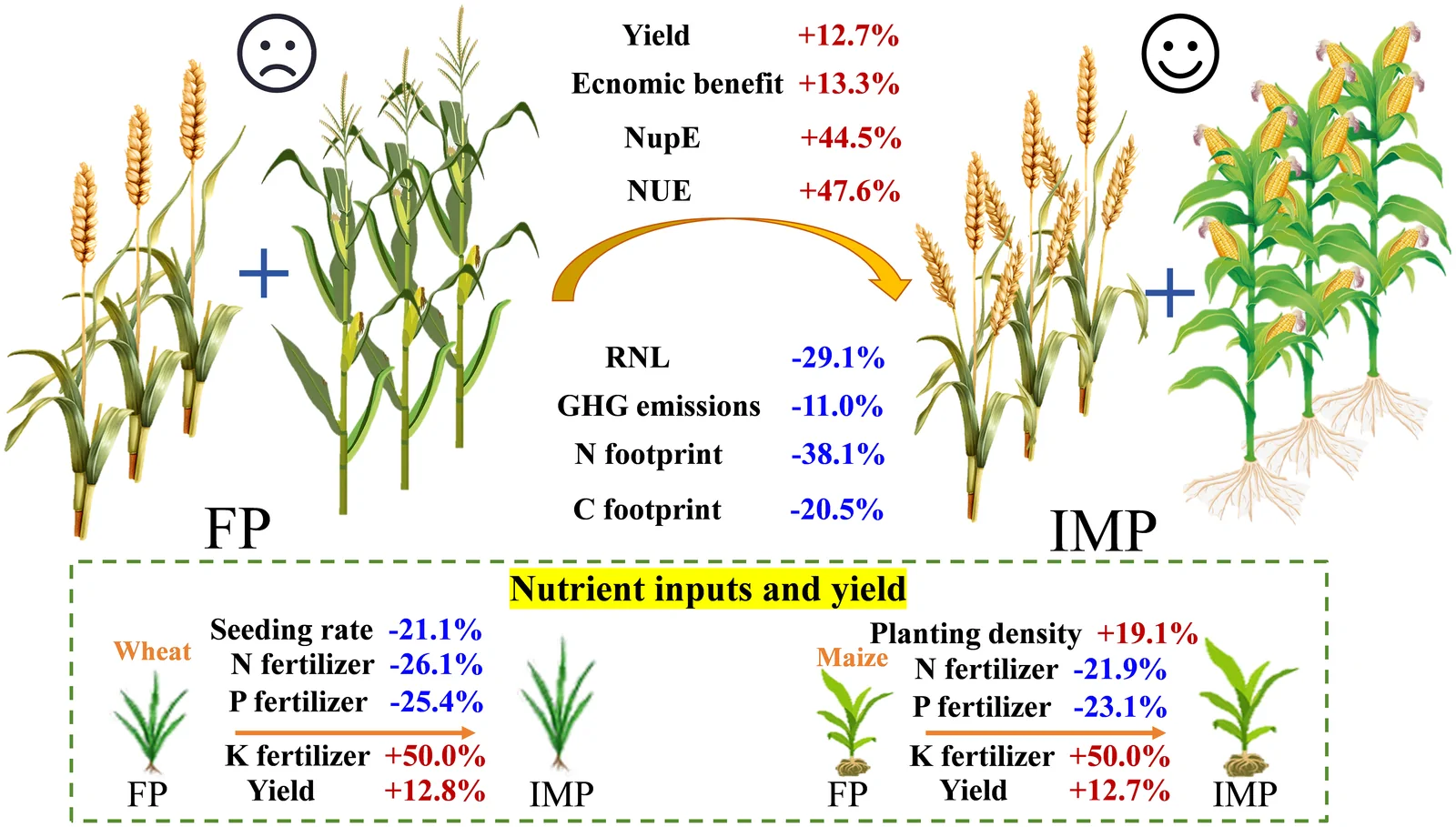
Effects of innovative management program on agronomic, economic benefits and environmental costs of the winter wheat/summer maize rotation cycle. FP represents farmer practices; IMP represents innovative management program. RNL, Reactive N losses; GHG emissions: greenhouse gas emissions; + denotes increase, - denotes decrease.

**Figure 9:**
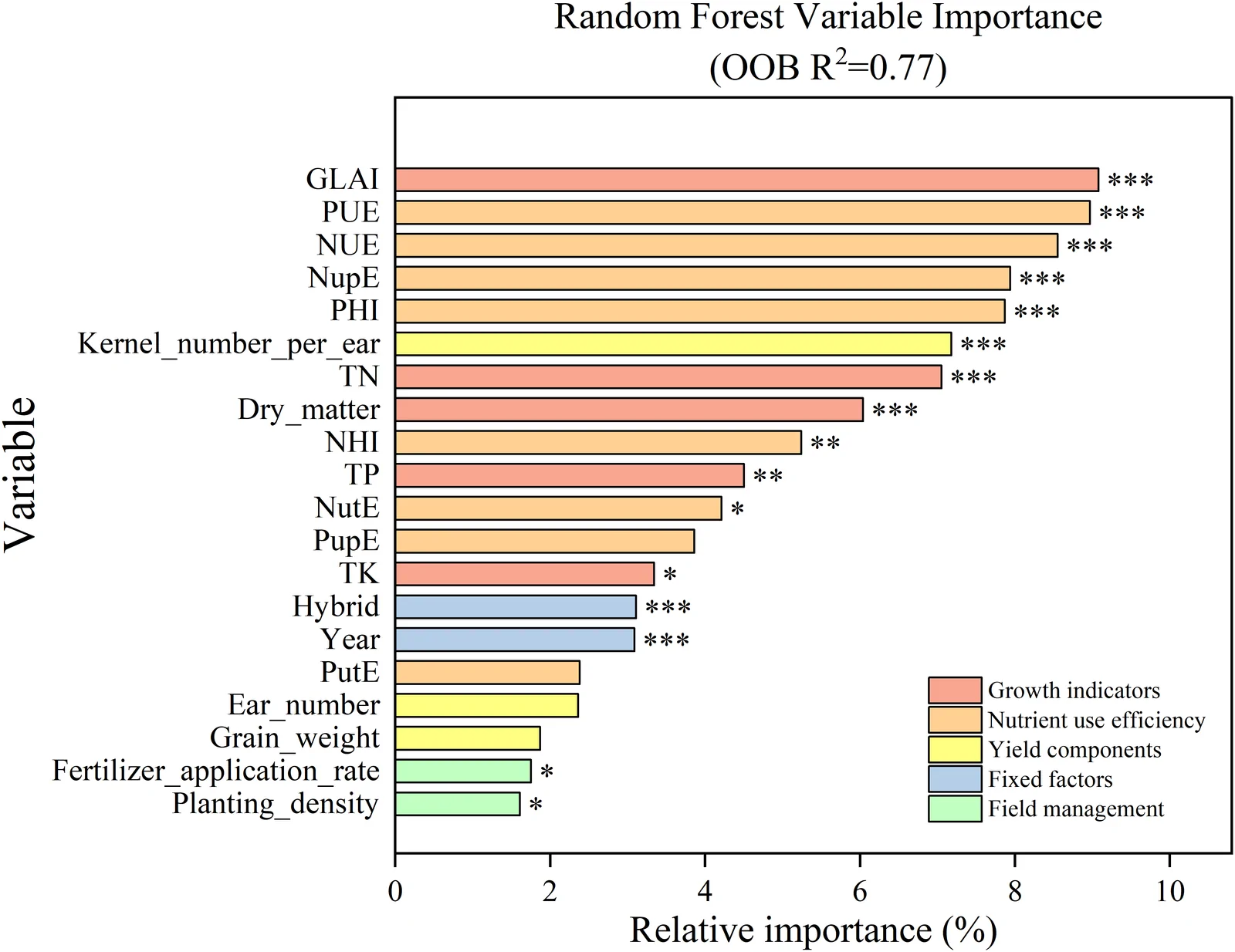
Random forest evaluation of indicator importance under different treatments.

**Figure 10:**
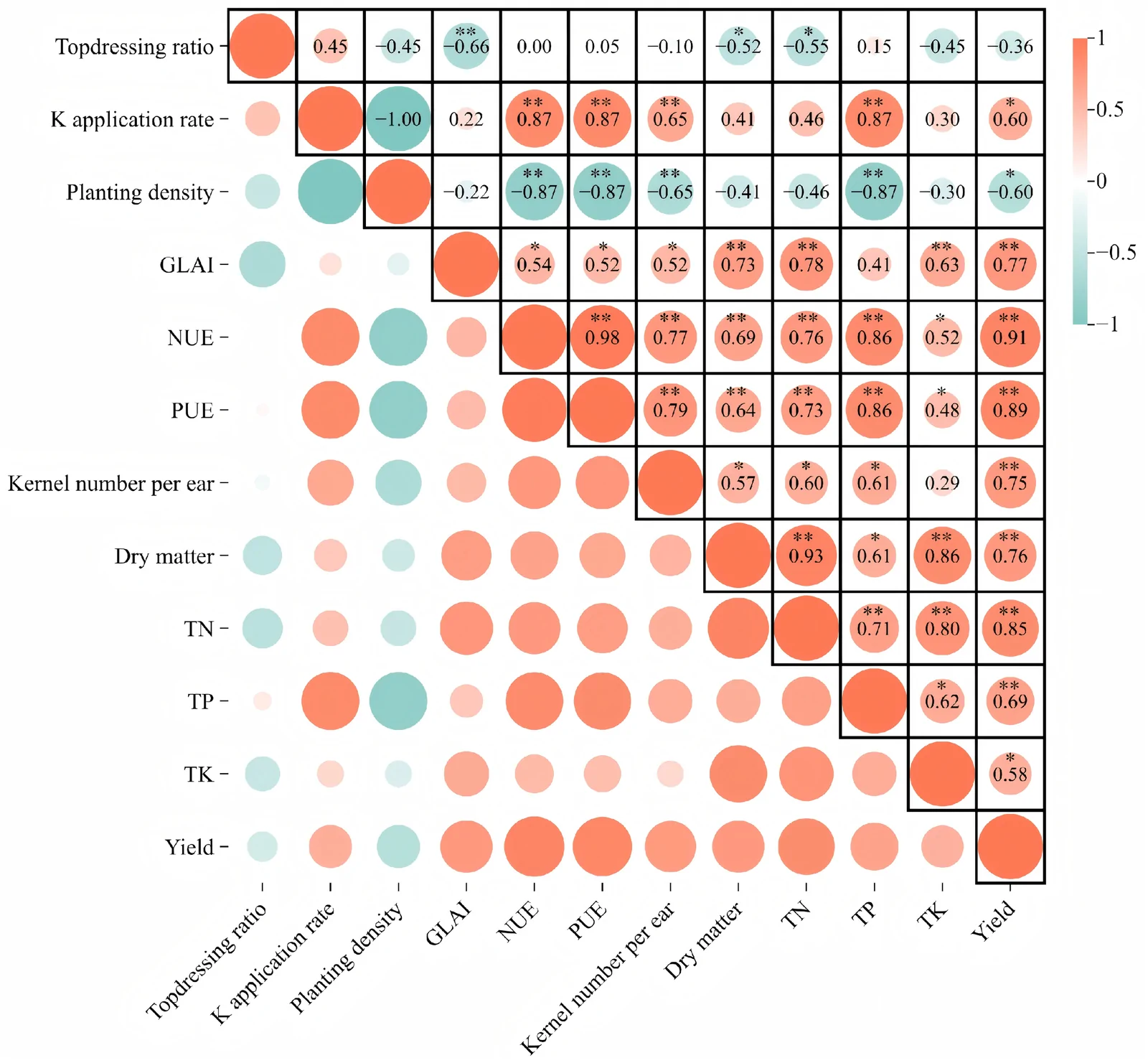
Pearson correlation heatmap between agronomic management practices and crop growth/yield-component traits. The color scale represents the correlation coefficient (r), with red indicating positive correlations and blue indicating negative correlations. * within the grid denote significant correlation levels (*P < 0.05, **P < 0.01).

### 3.4 Scenario analysis at village and regional (NCP) scales

Through the integrated application of the IMP treatment and the STB in Nanxia village, the winter wheat seeding rates and the application levels of N and P fertilizers decreased by 21.1%, 26.1%, and 25.4%, respectively, whereas the K fertilizer application and yield increased by 50.0% and 12.8%, respectively. The N and P fertilizer application levels for summer maize decreased by 21.9% and 23.1%, respectively, whereas the K fertilizer application level increased by 50.0%, resulting in a 12.7% increase in yield. In the winter wheat/summer maize annual rotation system, despite reducing N and P fertilizer application levels by 24.1% and 24.6%, respectively, the annual yield and income increased by 12.7% and 13.3%, respectively (**Figure 11**). The implementation of the IMP in Nanxia village drove a transition in winter wheat and summer maize production from a high-input, low-output system to one in which the implementation of scientific practices decreased excessive inputs, thereby cutting costs and increasing income.

**Figure 11:**
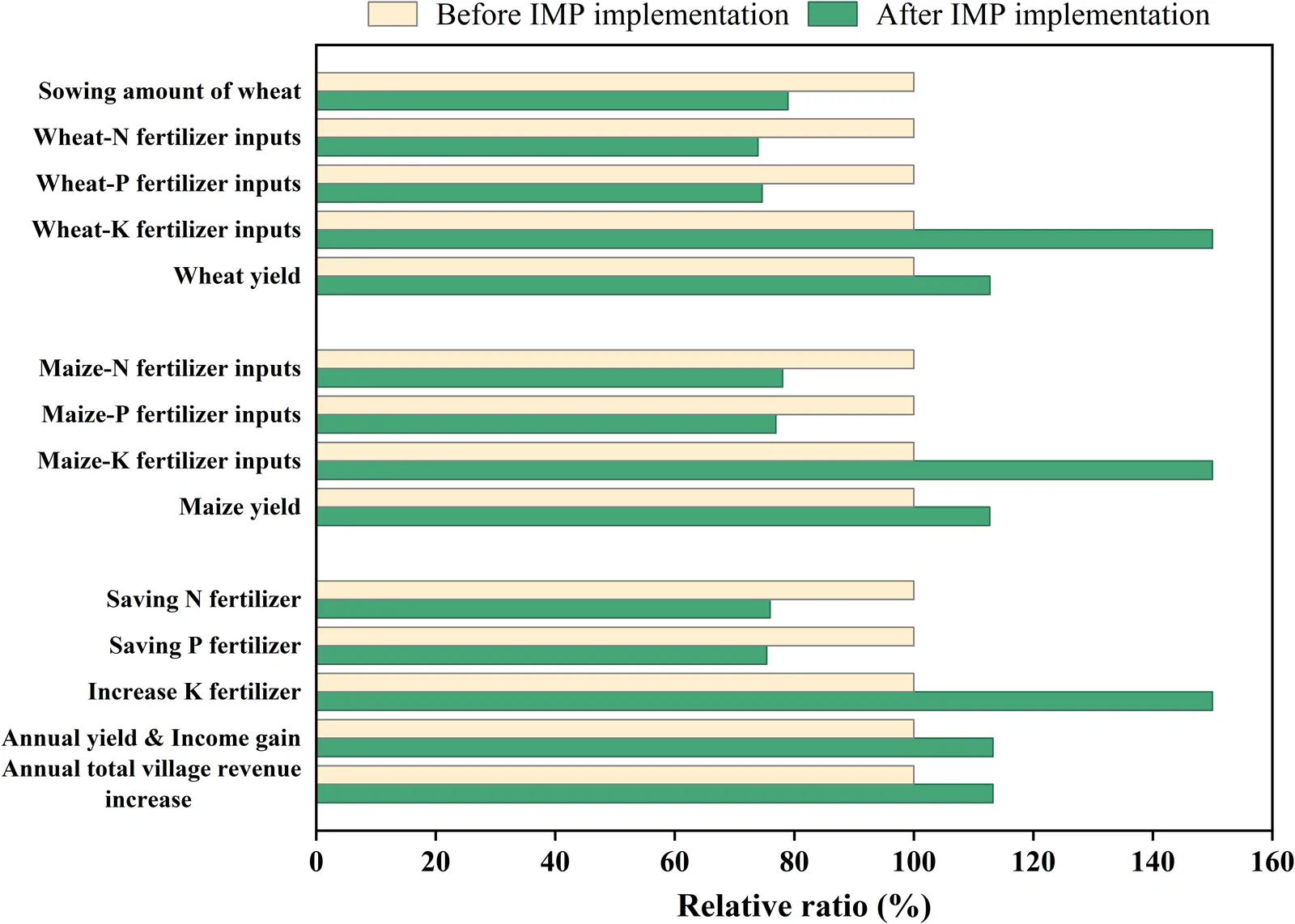
Effects of innovative management program on village improvement under winter wheat/summer maize rotation system. FP data are derived from a field survey (n=156); IMP data are from field experiment plots.

Assuming that the planting area remains unchanged, we defined two scenarios—Scenario 1 (IMP extended to 60% of the wheat/maize rotation area) and Scenario 2 (extended to 100%)—to explore the potential impacts of large-scale IMP adoption on the NCP. Owing to the uncertainties inherent in upscaling from site to regional scales, these scenarios project possible outcomes under idealized assumptions. Compared with those under the 2024 baseline scenario, the yield under scenario 1 increased 14.8 Mt, while the N and P fertilizer application levels decreased by 0.4 and 0.2 Mt, respectively. In Scenario 2, the yield increased by 24.6 Mt, with corresponding decreases of 0.8 and 0.3 Mt in the N and P fertilizer use, respectively. Moreover, the N_2_O emissions in Scenarios 1 and 2 decreased by 0.02 and 0.03 Mt yr^-1^, respectively, and the GHG emissions decreased by 3.3 and 5.4 Mt CO_2_-eq yr^-1^, respectively (**Table 4**). Therefore, the adoption of IMP technology in the wheat–maize rotation system on the NCP can simultaneously increase crop yields and reduce N_2_O and GHG emissions and fertilizer costs (**Figure 12**).

**Table 4:** Scenario analysis for the North China Plain.

Item	Unit	2024 Year	Scenario 1	Scenario 2
Area	Mha	32.8	32.8	32.8
Yield	Mt	193.8	208.6	218.4
N inputs	Mt	3.3	2.9	2.5
P inputs	Mha	1.0	0.8	0.7
N_2_O emissions	Mt N_2_O yr^-1^	0.14	0.12	0.11
GHG emissions	Mt CO_2_-eq yr^-1^	49.0	45.7	43.6

All data are derived from the National Bureau of Statistics (2025). Total N_2_O and GHG emissions are estimated by multiplying the published area-based emission rates by the corresponding cropping area. Scenario 1: The IMP treatment is adopted on 60% of the wheat–maize rotation area in the North China Plain. Scenario 2: The IMP treatment is adopted on 100% of the wheat–maize rotation area in the North China Plain.

**Figure 12:**
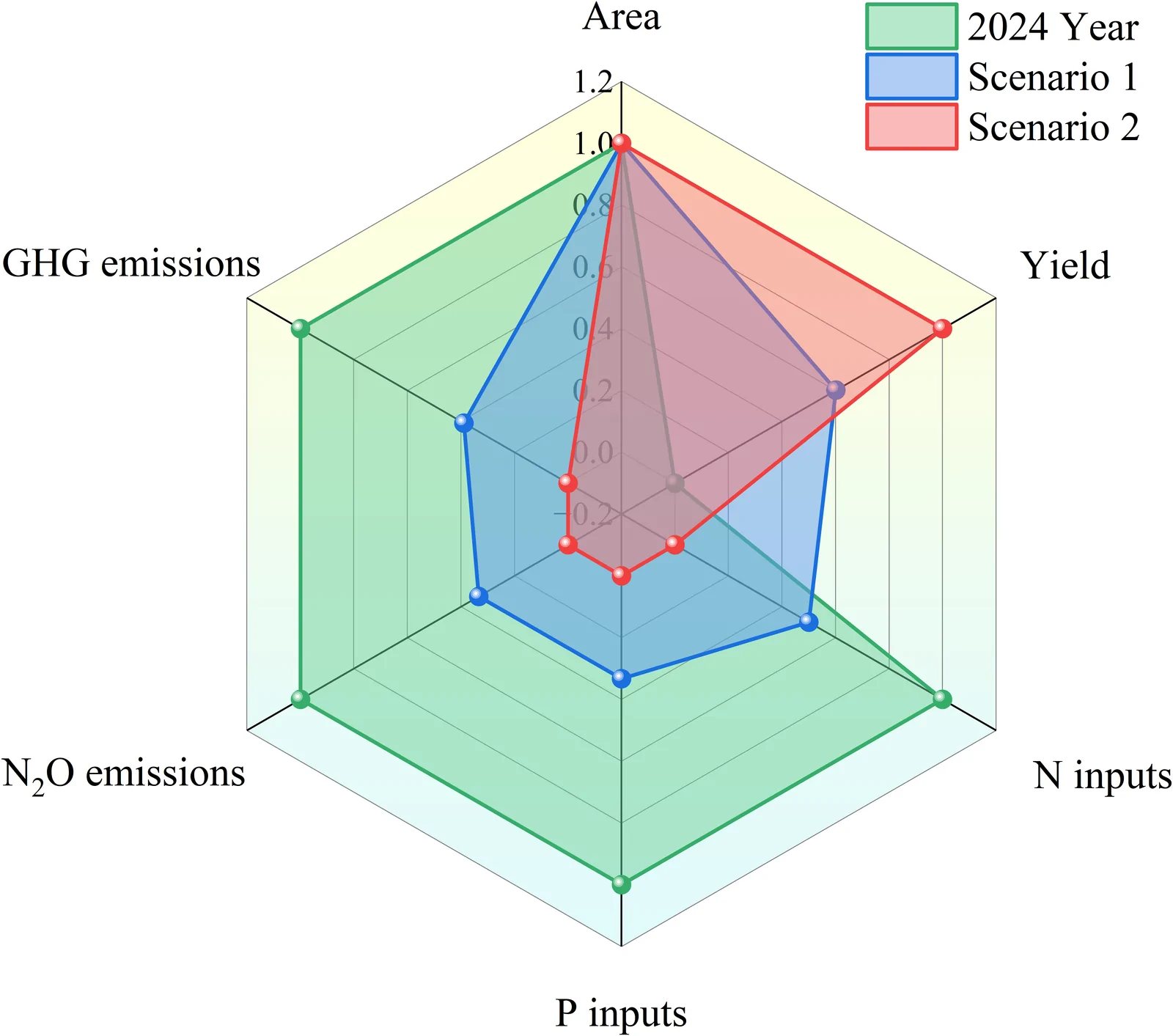
Effects of innovative management program on the North China Plain under winter wheat/summer maize rotation system.

## 4 Discussion

### 4.1 Effects of the innovative management program on winter wheat/summer maize yield and N and P use

Despite the continuous increase in crop yield potential, recent technological advances have contributed little to actual yield increases, resulting in a persistent and significant yield gap (Holman et al., 2024). Identifying the factors that influence yield constraints is crucial for better understanding yield gaps and determining new approaches to improve agronomic practices (Wang et al., 2023a). Smallholder farmers often increase seeding rates and N fertilizer inputs to pursue larger ear numbers (Wang et al., 2009), but this practice does not translate into a significant yield of kernels for winter wheat (Li et al., 2012). In this study, the IMP treatment systematically optimized yield components through the integrated application of key agronomic measures, including the selection of the density-tolerant hybrid DH605 and an appropriate increase in the planting density (**Table 1**). In the wheat/maize rotation system, the IMP treatment reduced N and P fertilizer inputs while increasing the yield by 12.7% and significantly increasing nutrient use efficiency, including NupE and PupE, NUE, and PUE (**Figure 1**; **Tables 2**, **3**). Nitrogen and potassium coapplication can regulate photosynthetic capacity, promote aboveground biomass growth, and increase nitrogen uptake and utilization efficiency (Zhao et al., 2026). In this study, a Pearson correlation heatmap revealed that increasing K fertilizer application could increase yield by increasing the NUE, PUE, kernel number per ear, and TP content. In contrast, increasing planting density significantly reduced the abovementioned indicators (**Figure 10**). This is attributed to intensified competition among individual plants for environmental resources under increased planting density, where canopy shading induced by high-density planting constrains the utilization of photosynthetically active radiation (Li et al., 2026). Additionally, topdressing ratio was negatively correlated with the GLAI, dry matter accumulation, and TN (**Figure 10**). This is attributed to the excessively high topdressing ratio restricting early vegetative growth and nitrogen accumulation, ultimately compromising yield formation. Therefore, the IMP treatment played an important role in increasing the yield and yield components.

The GLAI and leaf area duration (LAD) govern dry matter accumulation and yield formation (Hajirad et al., 2025; Li et al., 2022). A greater GLAI provides canopy structural optimization and promotes light–N synergy, thereby increasing the leaf photosynthetic capacity, i.e., the source potential (Borrás et al., 2004; Li et al., 2022). In this study, the increase in the yield of the wheat/maize rotation system was primarily attributable to the increase in the GLAI (**Figure 9**). The IMP treatment increased the maize GLAI through the application of a new compound fertilizer and the timely use of topdressing, thereby promoting dry matter accumulation and increasing N and P uptake (**Figures 2**-**5**). In 2021, moderate K fertilization significantly increased the GLAI in the IMP treatment group, thereby directly promoting dry matter accumulation (**Figures 2**, **3**). These findings agree with those of Song et al. (2023), who reported that sufficient water, N, and P enable K to increase maize leaf growth, photosynthesis, and dry matter accumulation. Moreover, optimized N application during grain filling delays leaf senescence, thereby extending the dry matter accumulation period and increasing yield (Lai et al., 2022). Random forest modeling identified GLAI and N and P use efficiency as important predictors of yield. Specifically, the enhanced uptake efficiency of these two nutrients improves their utilization efficiency, thereby increasing their overall use efficiency (**Figure 9**). Therefore, the IMP treatment increased the GLAI to promote dry matter accumulation and nutrient (N and P) uptake, whereas the application of a new compound fertilizer and the timely use of topdressing delayed leaf senescence and increased the source capacity. This source–sink synergy ultimately resulted in higher grain yields (Fan et al., 2026).

In China, the N fertilizer use efficiency of maize is only 35–40%, which is significantly lower than the internationally advanced level of 50–65% (Gheith et al., 2022). More than half of N fertilizers are lost from maize fields via runoff, ammonia volatilization, N oxide emissions, and leaching, whereas excessive P application causes soil P surplus and environmental losses through runoff (Liu et al., 2025b; Zhang et al., 2024b). Improving crop management practices can help maintain high levels of readily available soil nutrients (Pan et al., 2018). Soil available P and K are key factors limiting winter wheat and summer maize yields (Chen et al., 2018). In this study, optimizing the supply of N, P, and K in the IMP treatment increased soil available P and K at maturity in the wheat/maize rotation system but reduced the alkali-hydrolyzable N content in the 40–60 cm soil layer. These changes positively affect crop growth and soil health (Liu et al., 2023). However, single fertilizer application by smallholder farmers on the NCP often results in low NUE and late-season nutrient deficits, constraining maize yield (Wang et al., 2010). The core goal of NUE enhancement is to achieve spatiotemporal synchronization between the soil N supply and crop demand through optimized application rates and timing (Liang et al., 2019). Delaying N application until the peak demand period can increase the NUE of maize (Hou et al., 2019; Wang et al., 2010), whereas N application at later growth stages enhances tiller-to-heading conversion and the yield of wheat (Mossedaq and Smith, 1994; Xu et al., 2013). In terms of winter wheat production on the NCP, optimal management synergistically increases grain yield and NUE while reducing soil nitrite loss by nearly 50% (Lu et al., 2015; Wang et al., 2024a). In this study, we ensured an adequate late-stage nutrient supply by reducing the total N input, optimizing the fertilization ratio, and delaying topdressing to the jointing stage for winter wheat and the flare opening stage for summer maize. Compared with the FP treatment, the IMP treatment significantly increased the NUE of both crops and increased the level of N accumulation in maize (**Table 2**; **Figure 4**). Therefore, synchronizing the N supply with the crop demand through optimized fertilizer application timing and rates is key to increasing NUE and yield in the wheat–maize rotation system in the NCP.

### 4.2 Effects of the innovative management program on the environmental costs and economic benefits of winter wheat/summer maize

Agricultural land use is the primary source of ammonia (NH_3_) emissions, contributing approximately 90% to global volatilization (Ju et al., 2016). Effective management of agricultural emissions is crucial for achieving the 2060 C neutrality goal (Liang et al., 2021). However, smallholder farmers currently apply large amounts of N fertilizer, and more than 50% of the applied N is not taken up by aboveground crop parts, resulting in significant nutrient loss (Cassman et al., 2002; Ladha et al., 2005). This issue is especially prominent in the typical winter wheat/summer maize rotation system on the NCP, with the two crops yielding distinctly different environmental impacts (**Figure 7**). The concentration of rainfall during the summer maize season exacerbated nitrate leaching, resulting in a significantly higher RNL than that during the winter wheat season (**Figure 7a**). In contrast, precipitation during the winter wheat season can meet only 25–40% of its water requirements, necessitating one rotary tillage and three irrigation events, whereas summer maize requires only one irrigation event (Guo et al., 2019). Therefore, compared with summer maize, winter wheat produces higher GHG emissions because of its greater fuel and energy consumption (**Figure 7b**). To address these problems, the IMP treatment reduced N fertilizer inputs and optimized N management, leading to decreases of 24.0%, 29.1%, and 11.0% in the N application rate, RNL, and GHG emissions, respectively, and ultimately reducing the C and N footprints of the system by 20.5% and 38.1%, respectively (**Figure 7c, d**). Notably, the environmental costs in this study were estimated via LCA modeling, not direct field measurements. While the model has been locally parameterized with good applicability, some uncertainty remains (Cui et al., 2018). Therefore, the implementation of innovative management programs can synergistically reduce the N and C footprints while maintaining crop yields, providing a feasible pathway for the typical rotation system in the NCP to achieve low-C agriculture and C neutrality.

Agricultural economic efficiency is an important component of sustainable agricultural development (Yang and Chen, 2016). However, since 1978, the rapid increase in the use of chemical fertilizers, pesticides, and machinery in Chinese agriculture has challenged its sustainable development (National Bureau of Statistics, 2019). Optimizing N fertilizer application is crucial for ensuring sustainable N management because it directly addresses the dual issues of reduced economic benefits and environmental degradation caused by excessive N loss (Zhang et al., 2021b). Therefore, enhancing economic benefits and reducing environmental risks are key issues for the green and sustainable development of agriculture in the future. In this study, compared with the FP treatment, the IMP treatment increased the agricultural input costs by 10.1% through the use of a new compound fertilizer and topdressing, resulting in yield-driven increases in the annual total output value ranging from 18.3–20.5%. Considering both the costs and the output value, compared with those of the FP treatment, the net economic benefits of the IMP treatment increased by 12.0% and 14.3% during the 2020–2021 and 2021–2022 seasons, respectively (**Supplementary Table S1**). These findings demonstrate that the adoption of innovative management programs can effectively balance economic benefits with environmental sustainability, providing a viable technical pathway for green agricultural development in the region.

### 4.3 Scenario analysis at the village and regional (NCP) scales

The Science and Technology Backyard (STB) is an initiative conceived and deployed by China Agricultural University (CAU) to test an innovative approach to technology transfer aimed at supporting smallholder farmers (Zhang et al., 2016). The core approach of this program involves STB staff residing in villages and collaborating with farmers to conduct research, education, and extension activities centered on technology transfer. By integrating institutional knowledge with farmers’ practical experience—a model known as participatory innovation—yield constraints can be effectively overcome (MacMillan and Benton, 2014). Specifically, STB staff provide technical guidance and data recording, synthesize lead farmers’ improved technical performance and report it back to the research station to drive continuous innovation, thereby helping smallholders achieve higher economic returns while improving environmental management (Zhang et al., 2016). An STB-based survey in Nanxia village, Laoling city, revealed excessive fertilization, poor irrigation timing, and experience-based pest control as key constraints in the FP treatment, leading to high costs, low yields, and low resource efficiency (**Table 1**; **Figure 8**). Analysis of comprehensive village-level data demonstrated that the IMP treatment resulted in both higher yields and lower input-related environmental costs, indicating the successful deployment and adoption of this technological model across the entire village (**Figure 11**).

Although this study was conducted in only one village in Laoling city, its central finding—that the IMP treatment can achieve the dual goals of high productivity and low environmental impact—has broad applicability to smallholder farming systems across the North China Plain. As in the study area, the wheat–maize cropping system in the NCP faces the concurrent challenges of water conservation, yield improvement, and high NUE (Zhou et al., 2024). Consequently, this technological model is highly feasible for scaling up from village-level demonstration to regional application, providing a practical and viable example for the sustainable development of intensive wheat–maize systems in the NCP. However, owing to the uncertainties inherent in upscaling from site to regional scales, these scenarios project possible outcomes under idealized assumptions. Assuming that the planting arearemains unchanged, extending the IMP treatment to 60% of the rotational cropping area in this region is projected to increase grain production by 14.8 Mt while reducing N and P fertilizer inputs by 0.4 and 0.2 Mt, respectively. Scaling up to 100% coverage would increase the total yield by 24.6 Mt, with corresponding fertilizer savings of 0.8 Mt for N and 0.3 Mt for P (**Table 4**). Fertilizer application not only satisfies crop nutrient demands but also significantly modulates soil physicochemical properties, thereby affecting soil N accumulation and cropland GHG emissions (Zhang et al., 2021a). According to Xu (2018), the average N_2_O emissions in the NCP are 0.68 kg N_2_O-N ha^-1^ season^-1^ for wheat and 2.10 kg N_2_O-N ha^-1^ season^-1^ for maize, with annual GHG emissions from the wheat/maize rotation system reaching 1,492 kg CO_2_-eq ha^-1^. Extending the IMP treatment to 60% of the regional rotational cropping area is projected to reduce N_2_O emissions by 20,124 t N_2_O yr^-1^ and GHG emissions by 3.23 Mt CO_2_-eq yr^-1^ over the full rotation cycle. Scaling up to 100% coverage can decrease N_2_O emissions by 33,541 t N_2_O yr^-1^ and GHG emissions by 5.39 Mt CO_2_-eq yr^-1^ (**Table 4**). Therefore, promoting IMP implementation in the NCP wheat/maize rotation system can increase yields while reducing N_2_O and GHG emissions and fertilizer costs, highlighting optimized fertilization as a key strategy to balance food security, emission mitigation, and economic viability (**Figure 12**).

## 5 Conclusion

The implementation of an innovative management program increased the yield primarily by increasing the GLAI and improving N and P uptake and utilization. The results of the village-level scenario analysis further revealed that the IMP treatment can significantly increase yields and economic benefits while reducing nutrient inputs. Collectively, the results of this study demonstrate that the IMP treatment can achieve the dual goals of high productivity and low environmental impact in intensive wheat/maize systems in the NCP. This study was conducted under specific experimental conditions and lacks systematic observations of the long-term effects of the implementation of the proposed innovative management program on the evolution of soil quality and life-cycle GHG emissions across the NCP. Therefore, future research should aim to integrate multisite field trials and long-term monitoring to further validate the adaptability and stability of IMP technology under different scenarios.

## Data Availability

The datasets presented in this article are not readily available because The data that has been used is confidential. Requests to access the datasets should be directed to Yanling Chen, chenyanling21@163.com.
