# Supplementary material for: Innovative management program for achieving high yield and emission reduction: a case study of wheat/maize rotation production in the North China Plain
Source: Front Plant Sci. 2026 Sep 28;17:1952966. doi: 10.3389/fpls.2026.1952966 (PMC13655077; doi:10.3389/fpls.2026.1952966)
Supplement: Supplementary file 1 [file DataSheet1.docx]

Supplementary Material

# 1 Supplementary Tables

**Table S1. Effects of innovative management program on annual economic benefits of winter wheat/summer maize.**

| Year | Treatment | Total output value  (CNY ha^-1^) | Agricultural input costs  (CNY ha^-1^) | Net economic benefit  (CNY ha^-1^) |
| --- | --- | --- | --- | --- |
| 2020–2021 | FP | 50725 | 9360 | 41366 |
|  | IMP | 56635 | 10306 | 46329 |
| 2021–2022 | FP | 59078 | 9360 | 49719 |
|  | IMP | 67142 | 10306 | 56836 |

Note: CNY indicates Chinese yuan.

# 2 Supplementary Figures

**Fig. S1. Temperature and precipitation in the study area (2020-2022).**

**.**
